# Dual-Tasking Effects on Gait and Turning Performance of Stroke Survivors with Diabetic Peripheral Neuropathy

**DOI:** 10.21315/mjms2021.28.2.6

**Published:** 2021-04-21

**Authors:** Kamaruzaman Tajuddin, Maria Justine, Nadia Mohd Mustafah, Lydia Latif, Haidzir Manaf

**Affiliations:** 1 Centre for Physiotherapy Studies, Faculty of Health Sciences, Universiti Teknologi MARA, Puncak Alam, Selangor, Malaysia; 2 Department of Rehabilitation Medicine, Faculty of Medicine, Universiti Teknologi MARA, Sungai Buloh, Selangor, Malaysia; 3 Discipline of Rehabilitation Medicine, Faculty of Medicine, University of Malaya, Kuala Lumpur, Malaysia

**Keywords:** diabetes, dual task, gait, neuropathy, stroke

## Abstract

**Background:**

Stroke survivors depend on the unaffected leg during walking and standing. The presence of diabetic peripheral neuropathy (DPN) affecting both legs may further affect the postural balance and gait instability and increase the risk for falls in such patients. Thus, this study was conducted to investigate the effect of dual taskings on the gait and turning performance of stroke survivors with DPN.

**Methods:**

Forty stroke survivors were recruited (20 with DPN and 20 without DPN) in this cross-sectional study design. Instrumented timed up and go (iTUG) tests were conducted in three different tasking conditions (single task, dual motor and dual cognitive). APDM^®^ Mobility Lab system was used to capture the gait parameters during the iTUG tests. A two-way mixed analysis of variance was used to determine the main effects of gait performance on three taskings during the iTUG test.

**Results:**

Spatiotemporal gait parameters and turning performance (turning time and turning step times) were more affected by the tasking conditions in stroke survivors with DPN compared to those without DPN (*P* < 0.05).

**Conclusion:**

Stroke survivors with DPN had difficulty walking while turning and performing a secondary task simultaneously.

## Introduction

Dual-tasking or multitasking is present in almost all daily activities and requires a good cognition and physical function coordination. For example, during walking, a person may do other things simultaneously, such as using mobile phones, responding to traffic light changes when crossing the street and to the sound of a moving car or motorcycles (1). A previous study showed that the ability to perform multitasking activities is impaired in a population with neurological disorders that have affected the population’s gait and function (2). Another study conducted in stroke survivors revealed that dual-tasking may cause increased double support time and reduction of gait speed (3). This study concluded that the changes observed were of clinical significance for stroke survivors, as loss of gait speed of more than 4 m/min is considered as a big distance for the population that have difficulty in walking (3).

One of the complications of type 2 diabetes mellitus (T2DM) is the changes in executive functioning during balance and gait tasks (4). A certain degree of executive functioning is required for a person to complete his or her daily activities (5). Many of these activities include aspects of gait and balance (5). In individuals with T2DM, executive functioning is affected, and there is a reduced ability to maintain gait and balance, thereby increasing their risk of falls (4). As executive function affects dual-tasking ability, individuals with T2DM and diabetic peripheral neuropathy (DPN) are also affected by dual-tasking conditions. Recent study have shown that the walking speed of individuals with T2DM and DPN is slower by 22% when cognitive tasks are added to walking compared to that of age-matched healthy individuals (6). The average walking speed of individuals with T2DM and DPN during cognitive tasks also exceeds the fall cut-off score, which is considered as a high risk for fall (6).

Turning during walking is an important part of daily walking, especially for stroke survivors (7, 8). The added difficulty of dualtasking while turning has been reported to further deteriorate their turning performance (7, 8). People with diabetes also have turning deficits as compared to healthy controls (9). However, to date, there is paucity of evidence on the impact of DPN on the turning ability of stroke survivors in either single-tasking or dual-tasking.

DPN is a leading risk factor for falls, secondary to muscle weakness and sensory loss (10). Given that stroke survivors depend on the unaffected leg during walking and standing, the presence of DPN affecting both legs can potentially affect gait performance and postural balance. Therefore, this study aimed to investigate the effect of DPN on gait and turning performance during timed up and go (TUG) tests in stroke survivors in three tasking. The presence of DPN will cause the dual-tasking TUG, (motor or cognitive) to further deteriorate compared with stroke survivors without DPN.

## Methods

### Participants

Forty stroke survivors were recruited in this cross-sectional study design from a stroke rehabilitation clinic, government-funded hospital in Kuala Lumpur, Malaysia, using purposive sampling. A power analysis in which the authors used a previous study (11) that compared the TUG performance between healthy controls and individuals with stroke showed that an estimated sample of 40 participants would provide 90% power with a risk of type I error of 0.05. Twenty stroke survivors had DPN. The DPN diagnosis was made by a medical doctor using a combination of multiple tests of ankle reflex, vibration and temperature, and pinprick and 10 g monofilament pressure sensations at the distal halluces (12). The inclusion criteria included: i) participants who are at least 6 months post-stroke; ii) can walk independently without walking aids for 10 m; iii) can walk holding a glass full of water with the unaffected hand and iv) can follow three-step command and do simple arithmetic calculations. The exclusion criteria included: i) participants with more than one incidence of stroke or other neurological conditions; ii) other diseases that cause peripheral neuropathy except for T2DM; iii) severe musculoskeletal pain and problems, and iv) Montreal Cognitive Assessment (MoCA) score of < 26 (12). All participants signed an informed consent form that was approved by the Institutional Ethics Committee before their participation.

### Outcome Measures

All outcomes were measured by a similar assessor to minimise experiment bias. Demographic data of participants were obtained, followed by a measure of their cognitive ability using MoCA. MoCA consists of 16 items and 11 categories, such as visuospatial and executive functions, naming, memory, attention, language, abstraction and orientation. This test requires approximately 10 min with a maximum score of 30 points. Each category has their own scoring criteria. Compared to another cognitive test (the Mini-Mental State Examination [MMSE]), MoCA strength is the additional component of executive function assessment (13).

Functional balance performance was measured using the Berg balance scale (BBS) and their motor functioning was measured using the stroke rehabilitation assessment of movement (STREAM). BBS is a 14-item objective measure designed to assess static balance and fall risk in the adult population. BBS has an excellent internal consistency at 14, 30, 90 and 180 weeks post-onset of stroke (Cronbach’s alpha = 0.92–0.98) (14). BBS is a proven psychometrically sound measure of balance deficit in post-stroke (15). STREAM consists of 30 items assessing voluntary movement of the upper and lower limbs and the basic mobility of the participant. The maximum score for this tool is 70, with 20 for the upper limb, 20 for the lower limb and 30 for basic mobility. The test-retest reliability is good with intraclass correlation (ICC) of 0.96 (16).

The MicroFET (Hoggan Health Industries, USA) was used to measure muscle power of the lower limbs. This device showed a good test-retest reliability in chronic stroke survivors (ICC = 0.80–0.89) (17). It also has an excellent concurrent validity between hand-held dynamometers with known weights (18). This test was performed by assessing the muscle contraction strength of the hip flexor, knee flexor and ankle dorsiflexor muscles (19).

All participants performed instrumented TUG (iTUG) test under single and dualtasking conditions using the APDM^®^ Mobility Lab (Mobility Lab, APDM Inc., Portland, OR, USA). This test was used to determine the characteristics of spatiotemporal gait parameters during walking in stroke survivors with or without DPN. This system makes use of an inertial measurement unit to calculate trunk and lower limb movements. The APDM^®^ Mobility Lab sensors allow clinicians to perform unobtrusive gait assessments in a simple and quick manner (20). An inertia-based sensor (used by the APDM^®^ Mobility Lab system) has been shown to have excellent agreement with the gold standard of the accelerometer (21). Three opal sensors were positioned at each participant’s ankle and L5 level of the lower back.

### Testing Procedure

Three opal sensors were positioned at each participant’s ankle and at the L5 level of the lower back. Participants were instructed to perform the iTUG test. Participants were required to walk 3 m, turn around and walk 3 m back to the starting point. The iTUG test measured full body gait (legs and trunk), turning and postural stability. Each participant performed three different tasking conditions while conducting the iTUG test. The three tasking conditions were i) single task; ii) dual-motor tasks (iTUG test with motor component) and iv) dual-cognitive task (iTUG test with cognitive component). For a single task, participants performed the iTUG without any secondary task. In the dual-motor condition, participants had to hold a cup of water while performing the iTUG test, whereas in dual-cognitive condition, participants had to do a subtraction task while performing the iTUG test. The researcher averaged the two trials for each condition with a 3 min break between tasks. The task sequence was fully randomised.

### Statistical Analysis

Analyses were performed using SPSS statistical software (version 23.0 SPSS Inc., Chicago, IL, USA). Descriptive statistics and normality test for all variables were conducted. Independent *t*-test was used to compare the demographic data between the stroke survivors with and without DPN. To estimate the main effects of dual tasks on gait performance with the iTUG test, we conducted a mixed analysis of variance with group (stroke with DPN versus without DPN) as the between-subject variable and task (single versus dual motor versus dual cognitive) as the within-subject variable. Post-hoc tests were performed using the Bonferroni method to reduce the possibility of type I errors.

## Results

The stroke survivors with DPN group ranged from 40 to 72 years old of age, while stroke survivors without DPN ranged from 40 to 71 years old. The characteristics of the participants are presented in Table 1, as follows:

The comparison of BBS, MoCA, STREAM and the muscle power of the paretic side is presented in Table 2.

As shown in Table 3, the results show that there was a significant variation between group effect for all variables: time taken to complete iTUG test [*F* (1, 38) = 4.50; *P* = 0.042; partial η^2^ = 0.106], stride velocity [*F* (1, 38) = 12.15; *P* = 0.001; *η*^2^_p_ = 0.242], stride length [*F* (1, 38) = 6.79; *P* = 0.013; partial *η*^2^_p_ = 0.106], gait cycle time [*F* (1, 38) = 15.55; *P* = 0.001; *η*^2^_p_ = 0.265], time taken to complete turning [*F* (1, 38) = 5.56; *P* = 0.024; partial *η*^2^_p_ = 0.128] and turning step [*F* (1, 38) = 8.65; *P* = 0.006; *η*^2^_p_ = 0.185].

The dual-task conditions significantly affected the gait parameters (*P* < 0.001) and the effect was similar in both groups (condition × group interaction, *P* < 0.001). Post-hoc comparisons indicated that the dual-cognitive-task condition led to a significant increase in the time to complete the iTUG test, stride velocity, gait cycle time, time to complete the turn compared with single-task and dual-motor-task conditions (*P* < 0.001 for both) and a significant difference between single-task and dual-motor-task conditions (*P* < 0.001). However, stride length was not affected by the dual-motor-task condition (*P* = 0.146). In addition, there was no significant difference in step time to complete the turn occurred between single-task and dual-motor-task conditions (*P* = 0.961).

## Discussion

This study focused on comparing the dual-tasking effects on gait performance of stroke survivors with and without DPN. In the spatiotemporal components, there was a significant effect of dual-tasking on the gait performance of stroke survivors with and without DPN, and there were significant between-group differences across the groups of stroke survivors. For the turning component, there was also a significant effect of dual-tasking on the turning performance of both groups, but the between-group differences were only significant for two parameters of the turning component (turning time and step time before turning) in stroke survivors with and without DPN.

Our study found that dual-tasking has an effect on the gait performance of both groups of stroke survivors with and without DPN. Furthermore, stroke survivors with DPN have significant differences in the reduction of all spatiotemporal components and some of the turning components compared to stroke survivors without DPN. One of the possible explanation is that stroke survivors with DPN have a much more complex and challenging walking pattern compared to those without DPN (22). Moreover, they have reduced average knee angles, maximum hip and knee angles, and minimum ankle angle compared to stroke survivors without DPN (22).

One of the findings of our study is that stroke survivors with DPN took a longer time to complete the TUG test compared to stroke survivors without DPN, either in a single-task or dual-task condition. These results are consistent with another study that showed stroke survivors with DPN walk slower than those without DPN (23). The slower walking speed leads to reduced vertical force production in the stroke group with DPN (23). Stroke survivors with DPN were also demonstrated to have an asymmetry in the vertical force production between the paretic and non-paretic legs (23). Stroke survivors without DPN exhibited almost similar vertical force production between the paretic and non-paretic legs, while the paretic side in stroke survivors with DPN has much lower vertical force production than the non-paretic side, which could contribute to the slower walking speed (23).

Another finding is that dual-tasking affects the straight walking components of the TUG test. Dual-tasking increases iTUG time, stride length, stride velocity, cadence and gait cycle time. Additionally, the dual cognitive task has more effects compared to the dual motor task on TUG time, stride velocity and gait cycle time. In turning, the dual cognitive task also causes longer turning time and longer step time before turning. For that, it can be concluded that the dual cognitive task took more attention away from the stroke survivors with DPN compared to the dual-motor and single tasks.

As one of the explanations of the findings, a study has reported that mild to moderate stroke survivors with diabetes had poorer cognitive screening outcomes (24). One of the cognitive domains that were particularly involved was the executive function (24). Any changes in the executive function of a person may affect gait performance (5). Attention is considered to be a specific example of an executive function (25) and has a vital role in multi-tasking walking (5). Reduced executive function performance affects the activity of daily living and increases risk for post-stroke disability in stroke survivors (26).

In this study, stroke survivors with DPN had a lower MoCA score than stroke survivors without DPN. The lower score showed that stroke survivors with DPN had reduced cognitive level compared to those without DPN. There have been several proposals that could explain the relationship between diabetes and reduced cognitive function (27). One of these is that DM has an indirect effect through clinical or subclinical vascular disease (27). The cognitive decline could be due to the cerebral microvascular and macrovascular damage that is associated with DM (28). Abnormal insulin regulation may also have a more direct effect on the cognitive function (27). Amyloid-beta protein accumulation in the brain caused by high insulin levels (29), is an early sign of Alzheimer’s disease (30). History of diabetes also could cause reduction in brain volume and lesion to the white matter (31).

One major limitation of this study is that we did not include a healthy age-matched control group and another group of stroke survivor with T2DM without DPN. The comparison between stroke survivors with T2DM with and without DPN could provide another angle for this study. Another limitation is that data collection was conducted in a physiotherapy gymnasium, which may not pose enough challenges to the participants during the dual-task conditions.

## Conclusion

The findings from this study provide new insight especially for the population of stroke survivors with DPN. Dual-task training could be important for gait training in stroke survivors with DPN by reducing the number of fall incidences. Further studies are recommended to determine if the changes in the gait performance during dual-tasking are associated with fall history and identify the effects of dual-task training in stroke survivors with DPN.

## Figures and Tables

**Table 1:** Demographics of participants (*n* = 40)

Variables	Mean (SD)		*t*-statistic (df)	*P*-value

	Stroke survivors without DPN (*n* = 20)	Stroke survivors with DPN (*n* = 20)		
Age (years)	55.30 (8.72)	57.56 (9.99)	0.76 (38)	0.451
Height (cm)	165.90 (9.12)	163.10 (8.81)	0.98 (38)	0.329
Weight (kg)	70.77 (15.52)	73.28 (15.14)	0.52 (38)	0.607
BMI (kg/m^2^)	25.53 (4.08)	27.53 (5.08)	1.37 (38)	0.177
Years of stroke	2.00 (1.41)	3.00 (2.10)	1.76 (38)	0.085
Paretic side	right = 13 left = 7	right = 11 left = 9		

**Table 2:** Cognitive and motor function, functional balance and paretic muscle power of the participants

Variables	Stroke survivors	Mean (SD)	*t*-statistic (df)	*P*-value
MoCA	No DPN (*n* = 20)	27.95 (1.61)	2.19 (38)	0.035*
	DPN (*n* = 20)	26.45 (2.61)		
STREAM	No DPN (*n* = 20)	81.49 (12.15)	1.07 (38)	0.293
	DPN (*n* = 20)	77.43 (11.94)		
BBS	No DPN (*n* = 20)	51.45 (1.96)	2.23 (38)	0.032*
	DPN (*n* = 20)	50.10 (1.97)		
Hip flexors	No DPN (*n* = 20)	7.13 (1.83)	0.66 (38)	0.514
	DPN (*n* = 20)	6.71 (2.19)		
Knee extensors	No DPN (*n* = 20)	8.22 (1.94)	1.04 (38)	0.306
	DPN (*n* = 20)	7.60 (1.84)		
Ankle dorsiflexors	No DPN (*n* = 20)	6.73 (1.67)	1.43 (38)	0.162
	DPN (*n* = 20)	5.99 (1.61)		

* Note: indicates significant difference

**Table 3:** Gait parameters across three conditions between stroke survivors with and without DPN

Variables	Stroke survivors	Mean (SD)			Effects of condition		Effects of group	

		Single	Dual motor	Dual cognitive	*F*-statistic *(df1, df2)*	*P*-value	*F*-statistic *(df1, df2)*	*P*-value
iTUG (sec)	DPN (*n* = 20)	18.32 (3.2)	22.27 (3.54)	26.58 (5.57)	88.99 (2, 76)	0.001*	4.50 (1, 38)	0.042*
	Without DPN (*n* = 20)	16.09 (3.69)	19.7 (4.39)	23.58 (5.59)				
Stride velocity	DPN (*n* = 20)	0.53 (0.21)	0.47 (0.21)	0.38 (0.16)	48.64 (2, 76)	0.001*	12.15 (1, 38)	0.001*
	Without DPN (*n* = 20)	0.78 (0.22)	0.67 (0.23)	0.56 (0.18)				
Stride length	DPN (*n* = 20)	0.82 (0.21)	0.73 (0.24)	0.68 (0.19)	34.60 (2, 76)	0.001*	6.79 (1, 38)	0.013*
	Without DPN (*n* = 20)	0.97 (0.18)	0.87 (0.22)	0.85 (0.19)				
Gait cycle time	DPN (*n* = 20)	1.51 (0.21)	1.71 (0.33)	1.86 (0.35)	38.94 (2, 76)	0.001*	15.55 (1, 38)	0.001*
	Without DPN (*n* = 20)	1.31 (0.19)	1.35 (0.14)	1.62 (0.31)				
Turning (sec)	DPN (*n* = 20)	4.86 (1.71)	6.19 (1.92)	6.68 (2.27)	52.34 (2, 76)	0.001*	5.56 (1, 38)	0.024*
	Without DPN (*n* = 20)	3.94 (1.27)	4.74 (1.14)	5.36 (1.93)				
Turning (step)	DPN (*n* = 20)	7.52 (1.82)	9.28 (2.57)	9.18 (3.26)	21.23 (2, 76)	0.001*	8.65 (1, 38)	0.006*
	Without DPN (*n* = 20)	6.73 (1.26)	8.00 (1.62)	7.58 (2.24)				

* Note: indicates significant difference
